# Help-seeking behavior among community-dwelling adults with chronic pain

**DOI:** 10.1080/24740527.2019.1570095

**Published:** 2019-02-22

**Authors:** Elizabeth G. Mann, Elizabeth G. VanDenKerkhof, Ana Johnson, Ian Gilron

**Affiliations:** a School of Rehabilitation Therapy, Queen’s University, Kingston, Ontario, Canada; b School of Nursing and Department of Anesthesiology and Perioperative Medicine, Queen’s University, Kingston, Ontario, Canada; c Department of Public Health Sciences, Queen’s University, Kingston, Ontario, Canada; d Departments of Anesthesiology & Perioperative Medicine and Biomedical & Molecular Sciences, Kingston General Hospital, Kingston, Ontario, Canada

**Keywords:** chronic pain, help-seeking behavior, health care utilization, pain management, Canada

## Abstract

**Background**: Some individuals with chronic pain do not seek care. This decision may be due to characteristics of the individual, pain, and/or their health professional(s).

**Aims**: This study aimed to identify and compare features of individuals with chronic pain, their pain and general health, and their health care professional between community-dwelling adults who did and did not seek care.

**Methods**: Randomly selected adults were mailed a study questionnaire that screened for chronic pain (pain persisting ≥3 months) and asked about their general well-being (Short Form [SF]-36), pain location (body diagram), pain intensity and characteristics (Leeds Assessment of Neuropathic Symptoms and Signs), experiences with health care professionals (Chronic Illness Resources Survey), and visits made to health professionals over the past year. Respondents were categorized as help-seeking (≥1 visit in the past year) and non-help-seeking (zero visits in the past year).

**Results**: Six percent of respondents (44/696) were non-help-seeking. These respondents differed in individual, pain, and health care professional characteristics when compared to those who did seek care. Specifically, when other variables were controlled, non-help-seeking individuals were less likely to be male (relative risk [RR] = 0.39, 95% confidence interval [CI], 0.18–0.86), report comorbid conditions (RR = 0.46, 95% CI, 0.22–0.98), report being treated as an equal partner in decision making (RR = 0.40, 95% CI, 0.18–0.93), and rate their health care professional as important to their pain management (RR = 0.39, 95% CI, 0.18–0.85). They were more likely to use over-the-counter medication to manage their pain (RR = 2.52, 95% CI, 1.14–5.58).

**Conclusions**: Experiences with health professionals play a role in determining whether an individual manages his or her pain independently. Future research should explore the safety of those who do not seek care.

## RÉSUMÉ

**Contexte**: Certaines personnes souffrant de douleur chronique ne se font pas soigner. Cette décision peut être attribuable aux caractéristiques des individus, de leur douleur ou de leur(s) professionel(s) de la santé.

**But**: Cette étude avait pour but de répertorier et de comparer les caractéristiques de personnes souffrant de douleur chronique, de leur douleur et de leur état de santé général, ainsi que de leur professionnel de la santé, chez des adultes vivant dans la collectivité qui se sont fait soigner et ne se sont pas fait soigner.

**Méthodes**: Un questionnaire d’étude a été expédié par la poste à des adultes sélectionnés de manière aléatoire. Ce questionnaire cherchait à dépister la douleur chronique (douleur persistant ≥ 3 mois) et comprenait des questions sur leur bien-être général (SF-36), sur l’emplacement de leur douleur (diagramme du corps, intensité et caractéristiques de la douleur (*Leeds Assessment of Neuropathic Symptoms and Signs*) sur leurs expériences passées avec des professionnls de la santé (*Chronic Illness Resources Survey*), et sur leurs consultations auprès de professionnels de la santé au cours de la denière année. Les répondants ont été classés en deux groupes : ceux qui se faisaient soigner (une visite au cours de la dernière année) et ceux qui ne se faisaient pas soigner (aucune consultation au cours de la dernière année).

**Résultats**: Six pour cent des répondants (44/696) ne se faisaient pas soigner. Ces répondants étaient différents de ceux qui se faisaient soigner en ce qui concerne leurs caractéristiques individuelles, ainsi que les caractéristiques de leur douleur et de leur professionnel de la santé. Plus précisément, lorsque les autres variables étaient contrôlées, les personnes qui ne se faisaient pas soigner étaient moins susceptibles d’être des hommes (RR = 0,39, IC 95% = 0,18 – 0,86), de faire état de comorbidités (RR = 0,46, IC 95% = 0,2 – 0,98), de mentionner avoir êté traité comme un partenaire égal dans la prise de décision (RR = 0,40, IC 95% = 0,18 – 0,93) et de considérer que leur professionnel de la santé était important pour la prise en charge de leur douleur (RR = 0,39, IC 95% = 0,18 – 0,85). Ils étaient plus susceptibles d’utiliser des médicaments sans ordonnance pour prendre en charge leur douleur (RR = 2,52, IC 95% = 1,14 – 5.58).

**Conclusions**: Les expériences passées avec des professionnels de la santé jouent un rôle dans le choix d’un individu de prendre en charge sa douleur par lui-même. D’autres études devraient se pencher sur la sécurité de ceux qui ne se font pas soigner.

## Introduction

Help-seeking behavior has been defined as “a problem focused, planned behavior, involving interpersonal interaction with a selected health-care professional” (p. 286).^1^ Thus, an individual first identifies a problem that she or he perceives as requiring the assistance of a health care professional and then seeks help. Of those living with chronic pain, many will seek the help of one or more health professionals for diagnosis, pain management, and to reduce the effect of pain on their lives (e.g., improve sleep despite pain). There is a group, however, who do not seek care for pain. These individuals, termed “silent pain sufferers” by Watkins and colleagues,^2^ have been estimated to represent about 20%–67% of those reporting chronic pain in survey studies.^1–3^

Based on the above definition, there are three general elements of help-seeking that may explain why some individuals with chronic pain do not seek care: (1) characteristics of the affected individual (e.g., an expert self-manager may not require the help of a health care professional), (2) characteristics of the problem (e.g., mild pain that is not perceived to be a problem), and (3) characteristics of the health care professional and/or setting (e.g., office is located in such a way as to be inaccessible for the affected individual).^1^ Within chronic pain (the problem of interest in this article), prior research has identified that individual characteristics of those with chronic pain who are “silent” include young age, male gender, beliefs and attitudes about pain (e.g., belief that pain is not a sign of an underlying condition requiring treatment),^1,4,5^ beliefs and attitudes about doctors,^6,7^ and use of over-the-counter pain medications.^2^ Silent pain sufferers are less likely to report use of prescription medications and use of other pain management strategies (e.g., chiropractic interventions)^2^ to reduce pain. Pain characteristics of those not seeking care include pain located in the head or face, mild in intensity, low resulting disability, higher quality of life, and good general health.^1,2,5^ Conversely, these individuals are less likely to report pain located in the chest^2^ and social/familiar pressure to seek help.^1^ Sudden exacerbations have also initiated seeking care.^6^ There is some variation in reported findings in that studies have failed to consistently identify a relationship between gender, age, and pain intensity with help-seeking behavior.^1,4^ Older adults, for example, may be less likely to seek care if they hold to the view that pain is a normal part of aging.^3^ Characteristics of the health care professional have not been largely explored in the quantitative literature, but the limited results indicate that the way in which a health care professional is perceived by an affected individual can encourage or discourage his or her help-seeking behaviour. For example, affected individuals may be less likely to seek professional help if they have had a prior negative experience (e.g., perceiving that the health care professional did not listen to their concerns) or if they felt that their health care professional had a judgmental attitude.^1,7^ Qualitative research has added that vicarious negative experiences of loved ones and the assumption that the only help that health care professionals can provide is the prescription of medications also make seeking care less likely.^3,6^

The silence of this chronic pain group may or may not be a problem. It is expected that those individuals experiencing mild pain with minimal interference and having no other chronic conditions to monitor do not seek care. However, a proportion of this group reports severe pain, pain interference with sleep and general activity, and/or comorbid chronic conditions such as diabetes.^1,2^ For this group, their silence may be putting them at an increased risk of impaired workplace performance, self-care activity, disordered sleep, mental illness, suicide, death, social isolation, cardiovascular and gastrointestinal disease, and other health complications resulting from unmonitored and unmediated pain.^8,9^

Thus, this study seeks to generate hypotheses as to why some individuals with chronic pain do not seek care, expanding on what is known by including characteristics of the health care professional helping with pain management. The study objectives are to identify and compare features of the affected individuals and their health care professional and health problem (pain and general health) between community-dwelling adults who did and did not seek care over a 1-year period.

## Materials and methods

This study is a secondary analysis of a national survey of randomly selected, community-dwelling adults living in Canadian provinces. The main study explored the epidemiology of chronic neuropathic pain and how this pain was self-managed. Eight thousand telephone book addresses were mailed a survey cover letter and questionnaire with a unique study ID such that participant anonymity could be maintained. One follow-up questionnaire was mailed to nonrespondents. The cover letter detailed that consent was implied if the questionnaire was returned. To be included in this study, participants had to have responded positively to questions asking about the presence of pain and its persisting for at least 3 months^10^ and have at least partially completed the health care use items. More detail on the survey methods is available elsewhere.^11^ This study was reviewed for ethical compliance by the Queen’s University and Affiliated Hospital Research Ethics Board.

### Help-seeking

For this study, the above theoretical definition of help-seeking was operationalized as visits paid to a health professional. Help-seeking was captured with one four-part item that asked respondents to give the number of visits made to the following settings in the past year: family doctor, specialist, walk-in clinic, and emergency room. The goal was to examine help-seeking overall given the complexity of chronic pain; therefore, respondents were asked to report visits for any reason, not just for pain. Based on responses to this item, participants were categorized into one of two groups: help-seeking and non-help-seeking. A participant was considered to engage in help-seeking if she or he reported one or more visits to at least one of the above health care settings in the past year, as is consistent with similar studies.^12^ A second item captured visits to an “other” health care professional, but responses to this item have been analyzed and reported separately based on prior research suggesting different drivers for traditional and alternative care.^13^ Though some definitions of help-seeking include use of medications,^14^ the definition used in this article requires an interaction with a health care professional. Because it is unknown whether an individual interacted with a pharmacist, for example, use of medications was not considered to meet the definition of help-seeking.

### Individual characteristics

Individual characteristics were captured with a fill-in-the-blank for age and tick-box options for gender (male/female), employment status (nine options including working full or part time, retired, unemployed, unable to work, and other), and annual household income (five options ranging from <$19 000 to $150 000+). Pain beliefs were captured using the ten-item Pain Self-Efficacy Questionnaire,^15^ where respondents rated (0–6 scale) their confidence on statements such as, “I can live a normal lifestyle, despite the pain.” Responses are summed to generate scores ranging from 0 to 60; scores of 40 or greater were categorized as high self-efficacy and scores less than 40 were categorized as low self-efficacy.^16^ Testing supports the use of this tool as a valid and reliable measure of self-efficacy.^15,17,18^ Participants’ pain management strategies were captured in response to one item from the Level of Expressed Need questionnaire asking “What treatments or medications are you receiving for your pain?”^14^ Common responses were grouped into the following categories: prescription medication, over-the-counter medication, physical therapy and/or exercise, chiropractic and/or massage therapy, other, and nothing. In addition to pain management strategies, participants were asked about their level of satisfaction with their ability to manage their pain. One item from the Chronic Illness Resources Survey (CIRS) Family and Friend Scale was used as a general measure of the role of family and friends: “Have family or friends encouraged you to do the things you need to do for your illness?”^19,20^ This survey presents participants with five response options ranging from *not at all* (1) to *a great deal* (5). The CIRS has undergone psychometric testing with acceptable ratings of reliability and validity and correlation with other measures of social support.^19,20^

### Pain and general health problem characteristics

Pain characteristics were captured with four tools: a pain intensity numeric rating scale (NRS), the Self-Report Leeds Assessment of Neuropathic Symptoms and Signs Scale (SLANSS), one item on pain timing, and a body diagram for pain location. Participants were asked to indicate the intensity of their pain over the past week on an 11-point NRS, where 10 indicated highly intense pain and zero indicated no pain. NRSs have been identified as a quick, easy, and acceptable measure of pain that is suitable for self-administration.^21^ Ratings are recommended to be categorized as mild (0–4/10), moderate (5–7/10), and severe (8+/10) pain.^22^ The SLANSS was used as a screen for neuropathic characteristics. It is a seven-item tool that asks respondents about the characteristics of their pain, as well as the presence of related changes in sensation and appearance. Items are weighted based on the likelihood that a sign or symptom indicates a neuropathic component of pain and are summed to generate a total score ranging from 0 to 24. Scores of 12 or greater indicate that the pain has neuropathic characteristics.^23,24^ Though the usefulness of this tool in accurately identifying neuropathic pain appears to vary by pain diagnosis and use,^25,26^ it is considered a useful screening tool to identify neuropathic characteristics.^27^ Pain timing was captured with one item created for this survey that asked respondents to select whether their pain was experienced “all the time or daily,” “many days of the week,” “once per week,” or “once per month.” A body diagram with 35 delineated areas was presented to participants to capture pain location; participants were instructed to indicate all areas in which they experienced pain.^28^ The 35 areas were grouped into the following categories to provide adequate cell frequencies for analysis: head, face, or neck; back (including upper and lower back and spine); arms; hips;knees; and legs and/or feet.

The following were used to capture additional measures of respondents’ health problems: Short Form (SF)-36 to capture physical and mental aspects of health-related quality of life, the Patient Health Questionnaire-9 (PHQ-9) to capture depression, and one item to capture diagnosed chronic conditions. The items of the SF-36 capture health status information related to pain, physical and mental health, role and social functioning, health perceptions, and vitality are used in an algorithm provided by the instrument’s developers to generate two summary scores: mental and physical health component summaries.^29^ The reliability and validity of this widely used tool has been reported both during development^29–31^ and more recently in a variety of chronic conditions.^32–34^ The PHQ-9 is a nine-item tool that screens for the diagnostic criteria of depression listed in the *Diagnostic and Statistical Manual of Mental Disorders* (fourth edition).^35^ These criteria include decreased pleasure or interest in activities, difficulty concentrating, changes in sleep and/or eating habits, changes in activity levels and/or loss of energy, feelings of worthlessness and/or hopelessness, and thoughts of self-harm. Respondents report the frequency with which they experience the above symptoms with options ranging from *not at all* (0) to *nearly every day* (3). Testing of the tool indicates that categorizing scores of 10 or greater as indicating depression yields a specificity and sensitivity of 88% for major depression compared with diagnoses made via interviews by mental health professionals.^35,36^ This tool is widely available and is considered appropriate for use in ethnically diverse groups.^37^ The presence of comorbid chronic conditions was identified with one questionnaire item that asked, “Have you been told by a health professional that you have any of the following chronic health conditions?” Respondents were asked to select all that applied from the following list (adapted from the 2010 Canadian Community Health Survey^38^): asthma, anxiety disorder, bowel disorder, chronic respiratory disorder, chronic fatigue syndrome, diabetes, heart disease, hypertension, mood disorder, multiple chemical sensitivities, intestinal or stomach ulcers, stroke, urinary incontinence, or other.

### Health care professional characteristics

Participants were asked about the characteristics of their health care professional with the CIRS Physician and Health Care Team subscale, with one item asking about number of visits to an “other” health professional and one question about access to care. The CIRS subscale consists of seven items with response options ranging from *not at all* (1) to *a great deal* (5). Six of the seven items ask about the perceived actions of the health care professional with regards to the respondent and his or her chronic condition, and the seventh item asks the respondent how important the health care professional is to his or her self-management.^19,20^ Participants were instructed to consider their most recent experience with a health care professional when answering the CIRS items.

The role of access to health care services was elicited with one item in which respondents were asked whether access to health care services influenced their pain management.

### Analysis

Age, pain intensity (NRS), and both mental and physical component scores (SF-36; MCS and PCS) were normally distributed and are described with means and standard deviations and compared between the help-seeking groups (help-seeking and non-help-seeking) using *t* tests. Levene’s test of equality of variances was examined for *t* tests. The remaining continuous variables were categorized and described along with categorical variables using frequencies and percentages and compared between the two help-seeking groups with tests of relative risk (RR) and 95% confidence intervals (CIs). A binary logistic regression analysis was run for the purpose of generating hypotheses for future study. Variables significant at *P* < 0.20 in the unadjusted analysis were assessed for multicollinearity by examining variance inflation factors and tolerance statistics. With variance inflation factors < 2.50 and tolerance statistics > 0.40, these variables were entered into the equation in a hierarchical approach with individual characteristics as block 1, problem characteristics as block 2, and health care professional characteristics as block 3. Nonsignificant variables were removed in each block, starting with the least significant, until only significant variables remained. A sensitivity analysis was undertaken to determine whether grouping participants into three versus two groups changed the results. A multinomial logistic regression analysis was run comparing non-help-seeking individuals (zero visits) and those reporting one to three visits in the past year with those reporting four or more visits (reference group). Regression results are reported with odds ratios (ORs) and CIs. *P*-values have been included in addition to CIs for ease of interpretation; *P*-values < 0.05 were considered significant.

## Results

As reported in the larger study,^11^ 1509/8000 questionnaires were returned fully or partially completed. After adjusting for questionnaires returned with an error in the address (e.g., person had moved, incorrect address), this represents a 21.1% response rate (1509/7134). Despite instructions for the help-seeking item, participants frequently provided a numeric response for some of the settings and left others blank. Where a participant provided visit numbers for one or some of the four parts of the item, the participant remained in the analysis and blanks were considered to represent zero visits. If all four parts of them item were unanswered or a nonnumeric response was provided, the participant was dropped from the analysis. Using this method, 687 respondents reported chronic pain and completed the help-seeking item. Six hundred and fifty-four participants were categorized as help-seeking (reporting at least one annual health care visit) and 44 participants were categorized as non-help-seeking (6.3%).

### Individual characteristics

Response options for several variables were collapsed due to small cell frequencies, including education, annual household income, and pain satisfaction. After assessing skew and kurtosis values for the family and friend item of the CIRS, it was determined that responses to these items did not follow a normal distribution; thus, item responses were categorized as *not at all* (1–2/5) and *a moderate amount to great deal* (3+/5) to allow for comparison between the two groups. Forty-eight individuals indicated a medication that was illegible or unclear (e.g., “pain killers”) and thus could not be categorized as either over-the-counter or prescription. These responses were excluded from the analysis. There was no statistically significant difference between the mean age of participants in each group (help-seekers = 58.9 years vs. non-help-seekers = 55.2 years, *t* = −1.89, *P* = 0.06). Women (RR = 0.53, CI, 0.29–0.98), retirees (RR = 0.32, CI, 0.14–0.72), and those managing their pain with prescription medications (RR = 0.31, CI, 0.12–0.80) were less likely to be non-help-seeking (Table 1). Conversely, those using over-the-counter medications were twice as likely to be non-help-seeking (RR = 1.99, CI, 1.02–3.88). Participants in both groups reported similar ratings of satisfaction with ability to manage pain, with most participants being somewhat or fairly satisfied (help-seeking = 66.7%, non-help-seeking = 69.8%; RR = 1.12, CI, 0.53–2.39). In the adjusted analysis, only gender (OR = 0.39, CI, 0.18–0.86) and use of over-the-counter medications (OR = 2.52, CI, 1.14–5.58) remained associated with non-help-seeking behavior (Table 2).

**Table 1.** Individual characteristics of help-seeking and non-help-seeking participants.

Characteristic	Total (*n* = 696)	Help-seeking (*n* = 652)		Non-help-seeking (*n* = 44)		RR (95% CI)	*P*
	*n*	*n*	%	*n*	%		
Gender							
Male	352	323	91.8	29	8.2	1.00	
Female	342	327	95.6	15	4.4	0.53 (0.29–0.98)	0.04
Employment status							
Employed	334	306	91.6	28	8.4	1.00	
Retired	260	253	97.3	7	2.7	0.32 (0.14–0.72)	<0.01
Other	102	93	91.2	9	8.8	1.05 (0.51–2.16)	0.89
Annual household income $100 000+	162	147	90.7	15	9.3	1.00	
$50 000–$100 000	236	220	93.7	16	6.8	0.73 (0.37–1.44)	0.37
<$50 000	250	238	95.2	12	4.8	0.52 (0.25–1.08)	0.08
Pain self-efficacy							
High (40+/60)	502	465	92.6	37	7.4	1.00	
Low (<40/60)	157	152	96.8	5	3.2	0.43 (0.17–1.08)	0.07
Family and/or friends encourage pain management							
Not at all	176	162	92.0	14	8.0	1.00	
A moderate amount or great deal	483	458	94.8	25	5.2	0.65 (0.35–1.22)	0.18
Use of prescription medication^a^							
No	403	372	92.3	31	7.7	1.00	
Yes	207	202	97.6	5	2.4	0.31 (0.12–0.80)	<0.01
Use of over-the-counter medication^a^							
No	298	286	96.0	12	4.0	1.00	
Yes	324	298	92.0	26	8.0	1.99 (1.02–3.88)	0.04
Use of physical therapy and/or exercise							
No	550	520	94.5	30	5.5	1.00	
Yes	107	98	91.6	9	8.4	1.54 (0.75–3.15)	0.24
Use of chiropractic and/or massage therapy							
No	546	515	94.3	31	5.7	1.00	
Yes	112	104	92.9	8	7.1	1.26 (0.59–2.66)	0.55
Use of other pain management strategy							
No	517	484	93.6	33	6.4	1.00	
Yes	139	133	95.7	6	4.3	0.68 (0.29–1.58)	0.37
Use of nothing for pain							
No	585	552	94.4	33	5.6	1.00	
Yes	73	67	91.8	6	8.2	1.46 (0.63–3.36)	0.38
Satisfaction with ability to manage pain							
Completely satisfied or no significant pain	136	128	94.1	8	5.9	1.00	
Somewhat or fairly satisfied	455	425	93.4	30	6.6	1.12 (0.53–2.39)	0.77
Completely dissatisfied	89	84	94.4	5	5.6	0.96 (0.32–2.82)	0.93

^a^Forty-eight responses are missing due to the response being unclear with regards to medication type.

RR = relative risk; CI = confidence interval.

**Table 2.** Unadjusted and adjusted regression analyses of variables associated with non-help-seeking behavior.^a^

Variable	Total	Unadjusted analysis		Adjusted analysis^b^	
	(*n* = 696)	OR (95% CI)	*P*	OR (95% CI)	*P*
Gender					
Male	352	1.00		1.00	
Female	342	0.51 (0.27.–0.97)	0.04	0.39 (0.18–0.86)	0.02
Use of over-the-counter medication^c^					
No	298	1.00		1.00	
Yes	324	2.08 (1.03–4.20)	0.04	2.52 (1.14–5.58)	0.02
Comorbid chronic condition(s)					
No	288	1.00		1.00	
Yes	408	0.27 (0.14–0.53)	<0.01	0.46 (0.22–0.98)	0.04
Treated as equal partner by health care provider in decision making					
Not at all	264	1.00		1.00	
A moderate amount or great deal	382	0.32 (0.16–0.66)	<0.01	0.40 (0.18–0.93)	0.03
Ranked importance of health care provider to management					
Not at all	133	1.00		1.00	
A moderate amount or great deal	518	0.24 (0.12–0.48)	<0.01	0.39 (0.18–0.85)	0.02

^a^Nagelkerke *r*^2^ = 0.17.

^b^Entered into the equation (*P* < 0.20 in unadjusted analysis) were gender, employment status, annual household income, pain self-efficacy, family/friends encouraging pain management, use of prescription medication, use of over-the-counter medication, pain intensity, SF-36 PCS, SF-36 MCS, pain located in the back, pain located in the legs or feet, comorbid chronic conditions, HCP explained pain management, HCP treated participant as equal partner, HCP listened to concerns, HCP answered questions, HCP explained test results, ranked importance of HCP to pain management, and access to health care services.

^c^48 responses are missing due to the response being unclear with regards to medication type.

OR = odds ratio; CI = confidence interval; SF = Short Form; PCS = physical component score; MCS = mental component score; HCP = health care provider.

### Pain and general health problem characteristics

Pain timing options of “once per week” and “once per month” were rarely selected; thus, these options were collapsed into one category of “once per week or less.” There was no significant difference in how help-seeking and non-help-seeking individuals described their pain, with the exception of non-help-seeking individuals reporting slightly lower average pain intensities (NRS = 4/10 vs. 5/10, *t* = −2.50, *P* = 0.01) and being twice as likely to experience back pain (RR = 2.00, CI, 1.07–3.77; Table 3). Sixty-one percent of non-help-seeking participants (*n* = 27) rated their pain as mild in intensity, 31.8% (*n* = 14) rated their pain as moderate, and 6.8% (*n* = 3) rated their pain as severe. In contrast, 41.7% of help-seeking individuals (*n* = 268) rated their pain as mild, 43.1% (*n* = 277) rated their pain as moderate, and 15.2% (*n* = 98) rated their pain as severe (mild pain RR = 1.90, CI, 1.02–3.55). Better general health was also associated with non-help-seeking, because better scores of mental health (MCS = 51.4 vs. 48.7, *t* = 2.05, *P* = 0.04) and the presence of one or more comorbid chronic condition(s) (RR = 0.30, CI, 0.16–0.56) were associated with non-help-seeking behavior. However, after adjusting for other variables, only the presence of comorbid chronic conditions remained significant. Individuals with at least one comorbid had lower odds of non-help-seeking behavior (OR = 0.46, CI, 0.22–0.98; Table 2); that is, they were more likely to seek help than those without comorbid conditions.

**Table 3.** Pain and general health (problem) characteristics of help-seeking and non-help-seeking participants.

Characteristic	Total (*n* = 696)	Help-seeking (*n* = 652)		Non-help-seeking (*n* = 44)		Statistic	*P*
		Mean	SD	Mean	SD		
Pain intensity (NRS)	688	5.0	2.3	4.0	2.2	−2.50	0.01
SF-36 MCS	682	48.7	11.4	51.4	8.4	2.05	0.04
SF-36 PCS	682	42.0	9.8	44.5	9.1	1.62	0.11
	*n*	*n*	%	*n*	%	RR (95% CI)	*P*
Pain in head, face, or neck							
No	492	459	93.3	33	6.7	1.00	
Yes	204	193	94.6	11	5.4	0.80 (0.41–1.56)	0.52
Pain in back							
No	318	305	95.9	13	4.1	1.00	
Yes	378	347	91.8	31	8.2	2.00 (1.07–3.77)	0.03
Pain in arms							
No	337	315	93.5	22	6.5	1.00	
Yes	359	337	93.9	22	6.1	0.94 (0.53–1.66)	0.83
Pain in hips							
No	479	445	92.9	34	7.1	1.00	
Yes	217	207	95.4	10	4.6	0.65 (0.33–1.29)	0.22
Pain in knees							
No	434	409	94.2	25	5.8	1.00	
Yes	262	243	92.7	19	7.3	1.26 (0.71–2.24)	0.43
Pain in legs							
No	375	347	92.5	28	7.5	1.00	
Yes	321	305	95.0	16	5.0	0.67 (0.37–1.21)	0.18
Pain timing							
All the time or daily	335	318	94.9	17	5.1	1.00	
Most days of the week	258	239	92.6	19	7.4	1.45 (0.77–2.74)	0.25
Once per week or less	90	83	92.2	7	7.8	1.53 (0.66–3.58)	0.32
Neuropathic characteristics (SLANSS)							
No	494	462	93.5	32	6.5	1.00	
Yes	171	164	95.9	7	4.1	0.63 (0.28–1.41)	0.26
Comorbid chronic condition(s)							
No	288	257	89.2	31	10.8	1.00	
Yes	408	395	96.8	13	3.2	0.30 (0.16–0.56)	<0.01
Comorbid depression (PHQ-9)							
Unlikely	517	485	93.8	32	6.2	1.00	
Suspected	134	128	95.5	6	4.5	0.72 (0.31–1.69)	0.46

NRS = numeric rating scale; SF = Short Form; MCS = mental component score; PCS = physical component score; RR = relative risk; CI = confidence interval; SLANSS = Self-Report Leeds Assessment of Neuropathic Symptoms and Signs Scale; PHQ-9 = Patient Health Questionnaire-9.

### Health care professional characteristics

Similar to the above description of the family and friend scale, responses were categorized as *not at all* (1–2/5) and *a moderate amount to great deal* (3+/5) to allow for comparison between the two groups. Using this grouping, help-seeking and non-help-seeking respondents differed on all but one item on the CIRS Physician and Health Care Team subscale. Respondents who felt that their health care professional explained how to manage their pain (RR = 0.42, CI, 0.22–0.78), involved them as a partner in decision making (RR = 0.35, CI, 0.18–0.68), answered their questions (RR = 0.46, CI, 0.23–0.91), and explained test results (RR = 0.38, CI, 0.20–0.73) were less than half as likely to be non-help-seeking (Table 4). Respondents rating their health care provider’s importance in managing their pain as moderately to greatly important were 73% less likely to be non-help-seeking (RR = 0.27, CI, 0.15–0.50). Access to health care services was identified as an influence on pain management by 31.8% of non-help-seeking participants (*n* = 14) and 46.7% of help-seeking participants (*n* = 306; RR = 0.55, CI, 0.30–1.02).

**Table 4.** Health care professional characteristics of help-seeking and non-help-seeking participants.

Characteristic	Total (*n* = 696)	Help-seeking (*n* = 652)		Non-help-seeking (*n* = 44)		RR (95% CI)	*P*
	*n*	*n*	%	*n*	%		
Health care provider explained how to manage pain							
Not at all	199	180	90.5	19	9.5	1.00	
A moderate amount or great deal	450	432	96.0	18	4.0	0.42 (0.22–0.78)	<0.01
Treated as equal partner by health care provider in decision making							
Not at all	264	240	90.9	24	9.1	1.00	
A moderate amount or great deal	382	370	96.9	12	3.1	0.35 (0.18–0.68)	<0.01
Health care provider listened to concerns							
Not at all	107	97	90.7	10	9.3	1.00	
A moderate amount or great deal	544	518	95.2	26	4.8	0.51 (0.25–1.03)	0.06
Health care provider answered questions							
Not at all	110	99	90.0	11	10.0	1.00	
A moderate amount or great deal	542	517	95.4	25	4.6	0.46 (0.23–0.91)	0.03
Health care provider explained test results							
Not at all	114	101	88.6	13	11.4	1.00	
A moderate amount or great deal	529	506	95.7	23	4.3	0.38 (0.20–0.73)	<0.01
Ranked importance of health care provider to management							
Not at all	133	115	86.5	18	13.5	1.00	
A moderate amount or great deal	518	499	96.3	19	3.7	0.27 (0.15–0.50)	<0.01
Annual visit(s) to other health professional							
No	332	307	92.5	25	307	1.00	
Yes	363	344	94.8	19	344	0.70 (0.39–1.24)	0.22

RR = relative risk; CI = confidence interval.

In the adjusted regression analysis, both the perception of partnership between the participant and his or her health care provider (OR = 0.40, CI, 0.18–0.93) and perceived importance of the provider in management (OR = 0.39, CI, 0.18–0.85) reduced the odds of non-help-seeking behavior (Table 2).

### Sensitivity analysis

When the categorization of help-seeking groups was changed to no visits, one to three visits, and four or more visits, the presence of comorbid conditions and ranked importance of health care providers to pain management remained significant in the adjusted analysis for those reporting no visits (reference group = four or more visits) in the past year (Supplementary Table).

## Discussion

This secondary analysis explored the relationship between individual, pain problem, and perceived health care provider characteristics and seeking health care. Similar to prior research findings, both individual and pain problem characteristics played a role in seeking health care, with female gender, not using over-the-counter medication (individual characteristics), and the presence of comorbid conditions (pain problem characteristics) being associated with increased odds of seeking care. This study highlighted the role of perceived provider characteristics; specifically, those not seeking care in the past year had greater odds of feeling that their provider did not have a history of partnering with them or helping them manage their pain when other characteristics were controlled.

Despite participants in the help-seeking group having input from a health care professional and reporting slightly higher average pain intensity scores, they reported pain management strategies and use of “other” health care professionals similar to those of their non-help-seeking counterparts. Additionally, there was no difference in the proportion of individuals in each group who reported doing nothing to manage their pain. Use of prescription and over-the-counter medicine was the only point of difference noted in this study. The results of this study support prior findings that use of over-the-counter medication is associated with non-help-seeking behavior as well as the surprising finding that some participants report use of prescription medication despite not seeking care within the past year.^2^ Adding to these prior findings, the results of this study indicate that those who sought and did not seek health care in the past year reported similar rates of satisfaction with their ability to control their pain, with the majority of these individuals reporting that they are at least fairly satisfied. These results appear to describe an efficient use of the health care system in which individuals requiring prescription medication are followed by a health professional and those who are able to independently manage their pain with the use of over-the-counter medication do so and both groups are satisfied.

Participants in the help-seeking group reported using prescription medication, yet participants in both groups reported similar pain characteristics. Although the non-help-seeking group’s lower average pain intensity was statistically significant, it is debatable whether this one-point difference is clinically significant given that recommendations for outcome studies suggest a two-point or 30% reduction.^39^ This may account for why pain intensity did not remain significant after other study variables were controlled in the regression analysis. Though most non-help-seeking participants rated their pain intensity as mild or moderate, 6.8% (*n* = 3) rated their pain intensity as severe. Further analysis of this group was not possible due to the small number of non-help-seeking participants reporting severe pain in this study; however, this group would be an interesting subgroup to study in future research. Because the study questionnaire asked about visits to health professionals in general (not specifically related to pain), it is not surprising that the presence of at least one chronic condition was associated with help-seeking behavior. In prior studies that captured health care use specific to pain, the role of comorbid chronic conditions becomes nonsignificant.^13^

Differences between the two study groups were most apparent when they were compared on perceived health care professional characteristics. Though participants in both groups felt that their health care provider listened to their concerns, not seeking care in the past year was associated with lower ratings of perceived helpfulness of health care professionals in pain management.

This finding has also been identified in the qualitative literature, where individuals who felt their provider was unable to help them above their own self-management abilities did not seek care.^6^ It is unknown how non-help-seeking participants came to rate their care provider as unhelpful. Two possible explanations from qualitative research are (1) prior experiences with having their pain discounted^3,6^ and (2) only medication being offered for pain management.^3^ Individuals who felt that their pain was downplayed by health professionals cite being told that pain is an expected part of the normal aging process and thus does not require intervention (e.g., knee pain),^3,6^ feeling like their health care professional assumed that the cause was psychiatric, or feeling unbelieved if they did not have an identified cause of the pain.^40^ Taking reports of pain seriously, health care providers can begin the process of discussing pain management options with their clients. Some of the most commonly identified barriers to health care professionals suggesting adjunct use of nonpharmacological therapies revolve around a general lack of knowledge on both the part of the client and the provider.^41^ Increased provider education and client engagement in discussion of pain management options may improve the helpfulness of health care providers in pain management.

This study highlighted the potential importance of collaborative decision making and goal setting in help-seeking behavior. Gardner and colleagues compared common clinical goals with goals identified by individuals living with chronic low back pain, with the results suggesting that individuals present with a wide variety of personal therapy goals that extend beyond common clinical measures such as pain intensity.^42^ These personal goals included elements of coping, energy conservation, and building relationships. The potential breadth of personal therapy goals may explain why both help-seeking and non-help-seeking participants in this study reported similar pain, satisfaction with ability to manage pain, pain self-efficacy, and pain management strategies and yet diverged in ratings of their providers’ perceived helpfulness in pain management. With decision making and goal setting being the foci of recent research, a variety of strategies have been studied to guide the individual–provider dyad through the process from preparing to set a goal to following up on goal attainment.^43^

This study has several strengths and limitations. First, this study was a secondary analysis; thus, the questionnaire items were selected to address the aims of the original study. As such, participants reported total visits made over the past year rather than those made specifically for pain; thus, the role of comorbid chronic conditions could not be fully explored aside from including the presence of comorbid conditions in the regression analysis. This means of collecting visits did allow those reporting no help-seeking behavior in the past year to be analyzed separately rather than using tools that rely on respondent perceptions of what constitutes frequent seeking of care. The four-part item captured those who might be receiving care from a specialist or primary care physician or those without a primary care physician who rely on walk-in clinics or emergency rooms. This method also relied on participant recall over the preceding year, which, although thought to be a valid means of collecting health care use data,^44^ may have led to over- or underreporting of actual visits paid.^45,46^ This study focused on participants reporting no visits in the past year; as such, all other participants were grouped into a comparison group. The comparison group was not categorized into varying levels of health care use because this was not the focus of the current study and there are no set definitions to classify number of visits. In addition, other studies have examined differences between varying levels of health care use.^47^ Second, this study explored the role of the health care professional in help-seeking behavior, adding to what is known about the reasons why some individuals with chronic pain do not seek care. This analysis was limited, however, by the small proportion of participants reporting no visits to a health professional in the past year. These individuals may be less likely to participate in a study asking about chronic pain and its management; thus, the 6.3% categorized as non-help-seeking in this study may underestimate the true proportion of individuals with chronic pain who do not seek care. Due to this small sample size, the results of this study are limited in their application and are better suited to generating hypotheses for further study with powered sample sizes. This study assumed that all participants had seen a health care professional at some point in the past; however, non-help-seeking participants may not have seen a health care professional for a long time and thus their responses on the CIRS were also dependent on their ability to recall their most recent experience. Third, help-seeking behavior is known to be a complex and dynamic phenomenon, so despite capturing individual, problem, and health care professional characteristics, this study did not capture attitudes toward pain (e.g., stoicism^4^), duration of pain (e.g., newly diagnosed^48^), or pain-related disability^49^ beyond general health-related quality of life, which all likely contribute to help-seeking behavior. This may explain why the regression model explained only 17% of the variation in help-seeking behavior. In addition, this study was based on cross-sectional data; thus, though hypotheses can be generated, the causes of help-seeking cannot be determined.

Despite these limitations, this study has served to highlight the role of the health care professional in help-seeking and the need for these professionals to be cognizant of how their interactions with individuals may influence this behavior. For some individuals, support from a health care professional may not be necessary if self-management activities using readily available tools are enough to produce satisfaction with pain management. For others with more severe pain, a lack of collaboration in making decisions and setting goals may lead to the perception that health care professionals are unable or unwilling to help, leaving these individuals unsupported and vulnerable to the consequences of living with poorly treated pain.

### Implications

In light of similar reports of pain intensity, pain management strategies, and satisfaction with ability to manage pain, it appears as though the participants of this study found therapies that satisfied their pain management goals regardless of whether they had sought care in the past year. Currently, it is unknown whether this represents an ideal or unsafe situation. Further research is needed to determine whether those who do not seek care are at an increased risk of adverse events from both prescription and over-the-counter medications or other safety concerns. Because individuals may be most likely to seek care for a diagnosis at the onset of pain, health practitioners should consider self-management education a priority at these visits, including safe and effective use of over-the-counter medication. Conversely, it is unknown whether those who seek and receive the greatest *quantity* of health care experience the greatest *quality* of pain management or whether some instances represent an overuse of unnecessary testing and/or underuse of nonpharmacological management modalities.^49^ Determining what constitutes safe and cost-effective care for community-dwelling adults with chronic pain remains an ongoing challenge.

## Supplementary Material

Supplemental Material

## Supplemental Material

Supplemental data for this article can be access on the publisher’s website.
