# Supplementary material for: Help-seeking behavior among community-dwelling adults with chronic pain
Source: Can J Pain. 2019 Feb 22;3(1):8–19. doi: 10.1080/24740527.2019.1570095 (PMC8730570; doi:10.1080/24740527.2019.1570095)
Supplement: Supplemental Material [file UCJP_A_1570095_SM0856.docx]

Supplementary Table. Adjusted regression analysis of variables associated with no (n=44) and 1-3 visits (n=264) in the past year (reference = 4+ visits, n=391).

| Variable | 0 Visits | | 1-3 Visits | |
| --- | --- | --- | --- | --- |
|  | Odds Ratio (95% CI) | p | Odds Ratio (95% CI) | p |
| Use of Chiropractic and/or Massage Therapy  No  Yes | 1.00  1.94 (0.78 – 4.80) | 0.15 | 1.00  1.89 (1.14 – 3.14) | 0.01 |
| SF-36 Physical Component Score | 1.02 (0.98 – 1.06) | 0.42 | 1.06 (1.04 – 1.09) | <0.01 |
| Co-Morbid Chronic Condition(s)  No  Yes | 1.00  0.25 (0.11 – 0.55) | <0.01 | 1.00  0.33 (0.22 – 0.49) | <0.01 |
| Co-Morbid Depression (PHQ-9)  Unlikely  Suspected | 1.00  0.70 (0.27 – 1.84) | 0.47 | 1.00  0.41 (0.24 – 0.70) | <0.01 |
| Ranked importance of healthcare provider to management  Not at all  A moderate amount or great deal | 1.00  0.21 (0.10 – 0.47) | <0.01 | 1.00  0.73 (0.44 – 1.21) | 0.23 |
| Access to health services identified as influencing pain management  No  Yes | 1.00  0.67 (0.31 – 1.45) | 0.31 | 1.00  0.51 (0.34 – 0.76) | <0.01 |

Entered into the equation (p < 0.20 in unadjusted analysis) were: gender, employment status, annual household income, pain self-efficacy, family/friends encouraging pain management, use of prescription medication, use of over the counter medication, use of chiropractic and/or massage therapy, use of nothing for pain management, pain intensity, SF-36 PCS, SF-36 MCS, pain timing, co-morbid depression, co-morbid chronic conditions, health care provider (HCP) explained pain management, HCP treated participant as equal partner, HCP listened to concerns, HCP answered questions, HCP explained test results, ranked importance of HCP to pain management, and access to health care services.

Nagelkerke r^2^=0.31
